# Impacts of the Plateau Environment on the Gut Microbiota and Blood Clinical Indexes in Han and Tibetan Individuals

**DOI:** 10.1128/mSystems.00660-19

**Published:** 2020-01-21

**Authors:** Zhilong Jia, Xiaojing Zhao, Xiaoshuang Liu, Le Zhao, Qian Jia, Jinlong Shi, Xiao Xu, Lijun Hao, Zhenguo Xu, Qin Zhong, Kang Yu, Saijia Cui, Huining Chen, Jianying Guo, Xiang Li, Yang Han, Xinyu Song, Chenghui Zhao, Xiaochen Bo, Yaping Tian, Weidong Wang, Guotong Xie, Qiang Feng, Kunlun He

**Affiliations:** a Beijing Key Laboratory for Precision Medicine of Chronic Heart Failure, Chinese PLA General Hospital, Beijing, China; b Laboratory of Translational Medicine, Chinese PLA General Hospital, Beijing, China; c Key Laboratory of Biomedical Engineering and Translational Medicine, Ministry of Industry and Information Technology, Chinese PLA General Hospital, Beijing, China; d Ping An Health Technology, Beijing, China; e Shandong Provincial Key Laboratory of Oral Tissue Regeneration, Department of Human Microbiome, School of Stomatology, Shandong University, Jinan, Shandong, China; f State Key Laboratory of Microbial Technology, Shandong University, Qingdao, Shandong, China; g Beijing Institute of Radiation Medicine, Beijing, China; University of Waterloo

**Keywords:** gut microbiota, plateau environment, clinical indexes

## Abstract

The intestinal microbiota is significantly affected by the external environment, but our understanding of the effects of extreme environments such as plateaus is far from adequate. In this study, we systematically analyzed the variation in the intestinal microbiota and 76 blood clinical indexes among 393 healthy adults with different plateau living durations (Han individuals with no plateau living, with plateau living for 4 to 6 days, with plateau living for >3 months, and who returned to the plain for 3 months, as well as plateau-living Tibetans). The results showed that the high-altitude environment rapidly (4 days) and continually (more than 3 months) shaped both the intestinal microbiota and clinical indexes of the Han population. With prolongation of plateau living, the general characteristics of the intestinal microbiota and clinical indexes of the Han population were increasingly similar to those of the Tibetan population. The intestinal microbiota of the Han population that returned to the plain area for 3 months still resembled that of the plateau-living Han population rather than that of the Han population on the plain. Moreover, clinical indexes such as blood glucose were significantly lower in the plateau groups than in the nonplateau groups, while the opposite result was obtained for testosterone. Interestingly, there were Tibetan-specific correlations between glucose levels and *Succinivibrio* and *Sarcina* abundance in the intestine. The results of this study suggest that a hypoxic environment could rapidly and lastingly affect both the human intestinal microbiota and blood clinical indexes, providing new insights for the study of plateau adaptability.

**IMPORTANCE** The data presented in the present study demonstrate that the hypoxic plateau environment has a profound impact on the gut microbiota and blood clinical indexes in Han and Tibetan individuals. The plateau-changed signatures of the gut microbiota and blood clinical indexes were not restored to the nonplateau status in the Han cohorts, even when the individuals returned to the plain from the plateau for several months. Our study will improve the understanding of the great impact of hypoxic environments on the gut microbiota and blood clinical indexes as well as the adaptation mechanism and intervention targets for plateau adaptation.

## Abstract (precis)

The data presented in the present study demonstrate that the hypoxic plateau environment has a profound impact on the gut microbiota and blood clinical indexes in Han and Tibetan individuals. The plateau-changed signatures of the gut microbiota and blood clinical indexes were not restored to the nonplateau status in the Han cohorts, even when the individuals returned to the plain from the plateau for several months. Our study will improve the understanding of the great impact of hypoxic environments on the gut microbiota and blood clinical indexes as well as the adaptation mechanism and intervention targets for plateau adaptation.

## INTRODUCTION

High-altitude and hypobaric hypoxic environments, especially in areas >3,000 m above sea level, have great impacts on the physical and mental health of residents and travelers. Drastic changes in altitude induce acute mountain sickness (AMS) in newcomers and chronic mountain sickness (CMS) in long-term residents, which include headache, anorexia, insomnia, anxiety, and cerebral edema (1). The low oxygen level, low barometric pressure, and low temperature on the plateau results in decreased arterial oxyhemoglobin saturation, increased heart rate, increased blood pressure, and hormone disorders in Han individuals, while local Tibetans are unaffected. Human adaptation to the environment is closely related to the genetic background and acclimatization. For example, single nucleotide polymorphisms (SNPs) in EPAS1, SENP1, PPARGC1A, and EGLN1 were reported to be closely related to plateau adaptation (2–5).

The human gut microbiota is closely related to the immunological, hormonal, and metabolic homeostasis of human beings as well as various diseases and specific environmental adaptations. Moreover, the gut microbiota is an important indicator of human adaptation to the environment. Studies suggest that the high-altitude environment and gut microbiota collectively affect human beings physically and psychologically. Li and Zhao showed significant differences in the gut microbiota between Tibetan and Han individuals living on the plain and living in Tibet (6); specifically, the plateau-living Han individuals were enriched in relatively more of the phylum *Firmicutes*, while the plain-living Han individuals exhibited relatively high enrichment of microbes from the phylum *Bacteroidetes* (6). Li et al. also showed a significant difference in the intestinal microbiota between Tibetan and Han individuals, with *Prevotella* enriched in the Tibetans and *Bacteroides* being prevalent in plateau-living Han individuals (7). The altitude gradient-based gut microbiota study suggested that greater numbers of energy-efficient bacteria were enriched in the high-altitude group (7). The dominant gut bacteria in Tibetans living in different regions of the Qinghai-Tibet Plateau were *Bacteroidetes*, *Firmicutes*, *Proteobacteria*, and *Actinobacteria* at the phylum level, and facultative anaerobes prevailed in Tibetans (8). These studies suggest that altitude and culture significantly influence the composition of the gut microbiota; however, there is still no report on the changes in the microbiota and the physiological indicators for individuals entering and leaving the plateau. The changes in the clinical phenotype induced by a high-altitude, hypobaric, and hypoxic environment were probably related to stimulation and adaptation to the plateau. Liu et al. confirmed significant changes in heat rate, forced vital capacity, and mean flow velocity of the basilar artery in an AMS-susceptible cohort (9). The immune and inflammatory responses were disordered in the AMS individuals, and interleukin 10 (IL-10) was downregulated significantly. CMS is characterized by excessive erythrocytosis, exaggerated hypoxemia, and sometimes pulmonary hypertension (10), and various blood clinical parameters change markedly. The fasting blood glucose (GLU) level is relatively low in highland residents, as reported for residents in the Andes (11).

It is necessary to explore the clinical indexes for visitors with different living durations and local highland residents as well as the correlation between the gut microbiota and clinical indexes. In this study, we conducted a cross-sectional comparison between the gut microbiota and clinical indexes of Han individuals living on the plain (Han1k), those who stayed on the plateau for a few days (Han4k_4d and Han4k_6d) or for more than 3 months (Han4k), and those who returned to the plain for 3 months after >3 months of plateau living (Han4k_b3m) as well as those of local Tibetans on the plateau (Tibetan4k). The study will provide new research objects for mechanistic studies and intervention targets for plateau adaptation.

## RESULTS

**FIG 1:**
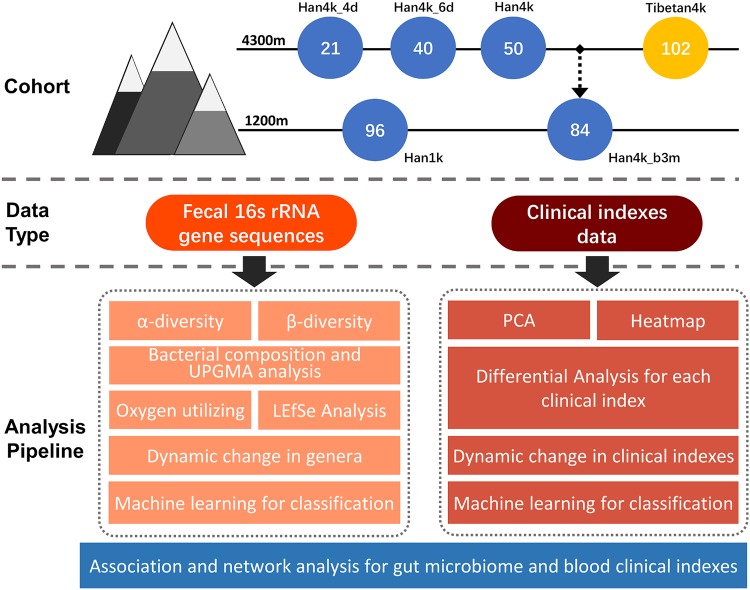
Overview of the analysis pipeline. The fecal and blood samples were collected and analyzed. The fecal samples were subjected to 16S rRNA gene sequencing followed by diversity analysis, bacterial composition analysis, functional prediction, and differential analysis. The blood samples were analyzed with 76 clinical indexes. The clustering analysis and statistical tests were applied to the clinical indexes. An XGBoost-based machine learning model was used to distinguish the different groups based on the bacterial and clinical indexes. Finally, analysis of the association and network between the gut microbiota and clinical indexes were used to illustrate the correlation and group-specific signatures.

Fecal and matched blood samples were collected from a total of 393 young male Chinese individuals, comprising 96 Han individuals (Han1k group) who lived on the plain, 21 Han individuals who stayed on the Tibetan Plateau for 4 days (Han4k_4d group), 40 Han individuals who stayed on the Tibetan Plateau for 6 days (Han4k_6d group), 50 Han individuals who stayed on the Tibetan Plateau for more than 3 months (Han4k group), 84 Han individuals who left the Tibetan Plateau and lived on the plain area for 3 months (Han4k_b3m group), and 102 native Tibetans (Tibetan4k group) whose families have lived on the plateau for generations. Notably, due to the unavailability of 37 blood samples, there were 356 matched blood samples, consisting of 83 Han1k, 18 Han4k_4d, 30 Han4k_6d, 48 Han4k, 75 Han4k_b3m, and 102 Tibetan4k samples. The detailed pipeline is shown in Fig. 1. More than 53 million raw reads from 16S rRNA gene sequencing were generated after filtering low-quality reads, and 50 million clean reads with lengths measuring approximately 450 bp were retained. Each sample covered, on average, 127,238 reads. The rarefaction curve analyses showed that saturation was reached in all samples (see Fig. S1 in the supplemental material). In total, 6,357 operational taxonomic units (OTUs), 740 genera, and 35 phyla were annotated in the SILVA database.

10.1128/mSystems.00660-19.1 FIG S1 (A) Rarefaction curve analyses of each group. (B) Group comparisons based on the Chao1 index. (C) Group comparisons based on the ACE index. (D) Group comparisons based on the Simpson index. (E) PCoA based on the weighted UniFrac distance of the microbial community between samples. (F) Weighted UniFrac distances from Han4k to other groups and from Han4k_b3m to other groups. Download FIG S1, TIF file, 1.2 MB. Copyright © 2020 Jia et al. 2020 Jia et al. This content is distributed under the terms of the Creative Commons Attribution 4.0 International license.

### Variations in the microbiota diversity influenced by high-altitude environments.

**FIG 2:**
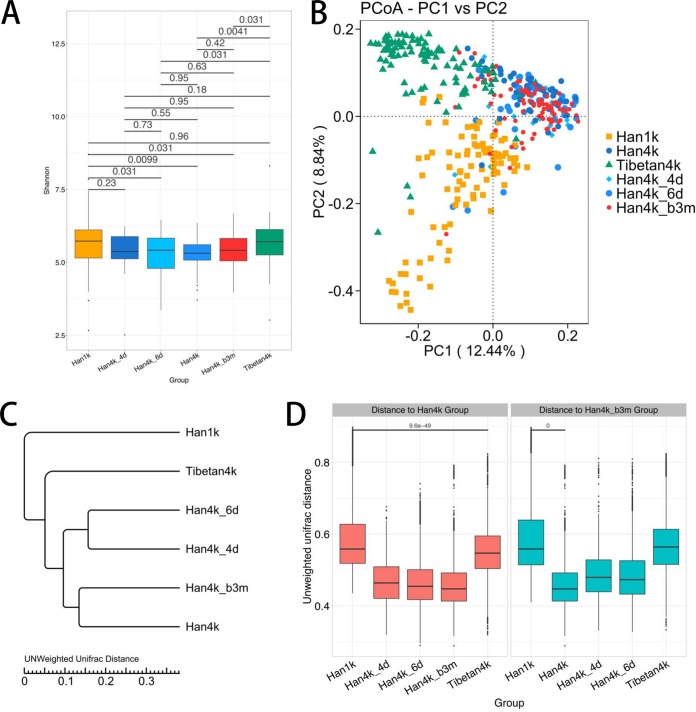
Diversity and distance of the microbial community in each group. (A) The Shannon indexes of the Han4k_6d, Han4k, and Han4k_b3m groups were significantly lower than those of the Han1k and Tibetan4k groups. The FDR-corrected *P* values shown were based on the Mann-Whitney U test with multiple-testing correction. (B) PCoA based on the unweighted UniFrac distances of the microbial communities between samples. The Han1k, Han4k, and Tibetan4k groups were representative and different from each other. Han4k_4d and Han4k_6d were closer to Han4k than to Han1k, and Han4k_b3m was closer to Han4k than to Han1k. (C) UPGMA tree based on the unweighted UniFrac distances between groups. (D) Unweighted UniFrac distances of different groups from the Han4k and Han4k_b3m groups. Statistical significance was labeled only for comparisons of interest.

An α-diversity analysis based on the Shannon index showed the differences among the 6 groups (Fig. 2A). The diversity of the intestinal microbiota in the short-term plateau-living Han populations (Han4k_6d) was significantly lower than that of the plain-living populations (Han1k) (false-discovery rate [FDR]-corrected *P* value = 0.031), and notably, the long-term plateau-living population (Han4k group) did not exhibit recovery (FDR-corrected *P* value = 0.95). Notably, the diversity of the intestinal microbiota hovered at relatively low levels for the Han4k_b3m compared with that for the Han1k individuals (FDR-corrected *P* value = 0.031). Interestingly, the difference between Han1k and Tibetan4k was not statistically significant (FDR-corrected *P* value = 0.96). The rarefaction curve and Chao1, abundance-based coverage estimator (ACE), and Simpson analyses also confirmed that the signatures among the different groups were robust (Fig. S1).

Principal-coordinate analyses (PCoAs) with unweighted UniFrac distances illustrated that Han1k, Han4k, and Tibetan4k presented three representative groups (Fig. 2B). Most of the Han4k_b3m population were also clustered near Han4k instead of Han1k. Notably, the majority of Han4k_4d and Han4k_6d were grouped with Han4k rather than Han1k. These groups were slightly separated in the PCoA graph based on the weighted UniFrac distance (Fig. S1). Each group was significantly different from the other groups based on Adonis analysis using Bray-Curtis dissimilarity (see Adonis result sheet in Table S1).

10.1128/mSystems.00660-19.9 TABLE S1 Clinical indexes sheet: all clinical index items and basic statistical descriptions between groups. Adonis result sheet: the result of the Adonis analysis for each group; bacterial composition sheet: descriptive statistics among groups for the top 10 bacteria at the phylum, family, and genus levels; LEfSe and Wilcoxon sheet: LEfSe and Wilcoxon results of the comparisons between Han1k and Han4k_b3m and between Han4k and Han4k_b3m (FDR-corrected *P* values were calculated by Wilcoxon statistical test with the Benjamini-Hochberg correction); genus groups sheet: a list of genera and their corresponding groups in Fig. 4; metadata statistics sheet: descriptive statistics among groups for age, weight, height, and BMI; BugBase sheet: Wilcoxon statistical test of aerobic and anaerobic bacteria at the phylum level in each group; Spearman sheet: all the Spearman correlation coefficients, *P* values, and FDR-corrected *P* values between genera and clinical indexes in each group. Download Table S1, XLSX file, 0.1 MB. Copyright © 2020 Jia et al. 2020 Jia et al. This content is distributed under the terms of the Creative Commons Attribution 4.0 International license.

The results of unweighted pair-group method with arithmetic means (UPGMA) tree analysis showed that Han4k was closer to Tibetan4k than to Han1k and further validated the results of PCoA (Fig. 2C). Furthermore, the unweighted and weighted UniFrac distance of each group to those of Han4k and Han4k_b3m also confirmed the results described above (Fig. 2D and S1). These results collectively suggest that the plateau environment has a profound impact on the intestinal microbiota of individuals who live on the plateau for a short or long period of time, and this effect may persist for quite a long time even after plateau living.

### Differences in microbiota composition between Han and Tibetan individuals.

**FIG 3:**
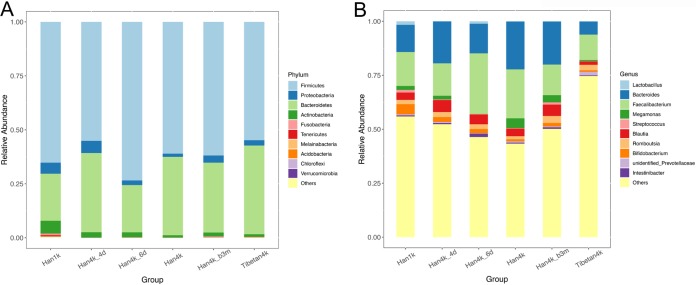
Bacterial composition in each group. (A) Relative abundances of the top 10 phyla. (B) Relative abundances of the top 10 genera.

The 10 most abundant phyla in each group are illustrated in Fig. 3A. Compared with that in the Han1k group, the relative abundance of *Proteobacteria* and *Actinobacteria* was significantly decreased in the Han4k, Han4k_b3m, and Tibetan4k groups (all FDR-corrected *P* values ≤ 0.007, all effect sizes [ESs] ≥ 1.5) (see Fig. S2 and bacterial composition sheet in Table S1). The relative abundance of *Bacteroidetes* was the lowest in the Han1k group compared with that in the other groups (all FDR-corrected *P* values < 0.001) (Fig. S2). The ratio of the relative abundances of *Firmicutes* and *Bacteroidetes* (F/B ratio) was the highest (9.83) in the Han1k group and lowest (2.06) in the Tibetan4k group. The F/B ratio of the Han4k group was more similar to that of the Tibetan4k group (FDR-corrected *P* value = 0.10, ES = 1.45) than to that of Han1k (FDR-corrected *P* value < 0.001, ES = 0.30), and the F/B ratio of the Han4k_b3m group was more similar to that of the Han4k group (FDR-corrected *P* value = 0.19, ES = 1.87) than to that of Han1k (FDR-corrected *P* value = 0.0013, ES = 0.56) (Fig. S2).

10.1128/mSystems.00660-19.2 FIG S2 (A) Statistical significance of bacterial abundance between groups in three phyla, namely, *Proteobacteria*, *Bacteroidetes*, and *Actinobacteria*. (B) Statistical significance of bacterial abundance between groups in four genera, namely, *Bacteroides*, *Faecalibacterium*, *Megamonas*, and *Bifidobacterium*. (C) Statistical significance of the ratio of the relative abundances of *Firmicutes* and *Bacteroidetes* (F/B ratio) between groups. Download FIG S2, TIF file, 0.8 MB. Copyright © 2020 Jia et al. 2020 Jia et al. This content is distributed under the terms of the Creative Commons Attribution 4.0 International license.

At the phylum level, the abundance analysis showed that the *Bacteroidetes* abundance was higher in the Tibetan4k group than in all the Han groups (1.13 ≤ ES ≤ 1.89) (Fig. 3A and S2). Compared with that in Han1k, at the genus level, *Bacteroides*, *Faecalibacterium*, and *Megamonas* were significantly enriched in Han4k (FDR-corrected *P* values < 0.025; ESs = 1.76, 1.45, and 2.5, respectively) and then depleted in Han4k_b3m (compared with Han4k, FDR-corrected *P* values = 0.219, < 0.001, and 0.043; ESs = 0.90, 0.63, and 0.73, respectively) (Fig. 3B and S2 and bacterial composition sheet in Table S1). Moreover, the abundances of *Bacteroides*, *Faecalibacterium*, and *Megamonas* genera were higher in almost all of the Han groups than in the Tibetan4k group. The abundance of *Bifidobacterium* in the Han groups decreased considerably (FDR-corrected *P* value < 0.001, ES = 0.21), recovering slightly (FDR-corrected *P* value = 0.006, ES = 1.6) with the changes in the states of the participants (Fig. S2 and bacterial composition sheet in Table S1).

The differences between groups were determined by both linear discriminant analysis effect size (LEfSe; *P* value < 0.05 and the logarithm of linear discriminate analysis [LDA] > 2) and the Wilcoxon test (FDR-corrected *P* value < 0.05) at the genus level. The common results of the two methods were considered differentially abundant bacteria. Compared with that in Han1k, 17 genera were increased and 68 genera were decreased in Han4k as a result of plateau living (see Fig. S3). The same analysis was performed for Han4k and Tibetan4k, and 71 genera were significantly changed between these groups (Fig. S3). The LEfSe and Wilcoxon test results of the comparisons between Han1k and Han4k_b3m and between Han4k and Han4k_b3m are shown in LEfSe and Wilcoxon sheet in Table S1. The change in the intestinal microbiota of Han individuals compared to that of the Tibetan individuals may be closely related to the plateau environment.

10.1128/mSystems.00660-19.3 FIG S3 (A) Eighty-five genera selected by LEfSe and the Wilcoxon statistical test between Han1k and Han4k groups. (B) Seventy-one genera selected by LEfSe and the Wilcoxon statistical test between Han4k and Tibetan4k groups. The genera are shown on the *y* axis and logarithmic LDA scores are shown on the *x* axis. Download FIG S3, TIF file, 0.4 MB. Copyright © 2020 Jia et al. 2020 Jia et al. This content is distributed under the terms of the Creative Commons Attribution 4.0 International license.

**FIG 4:**
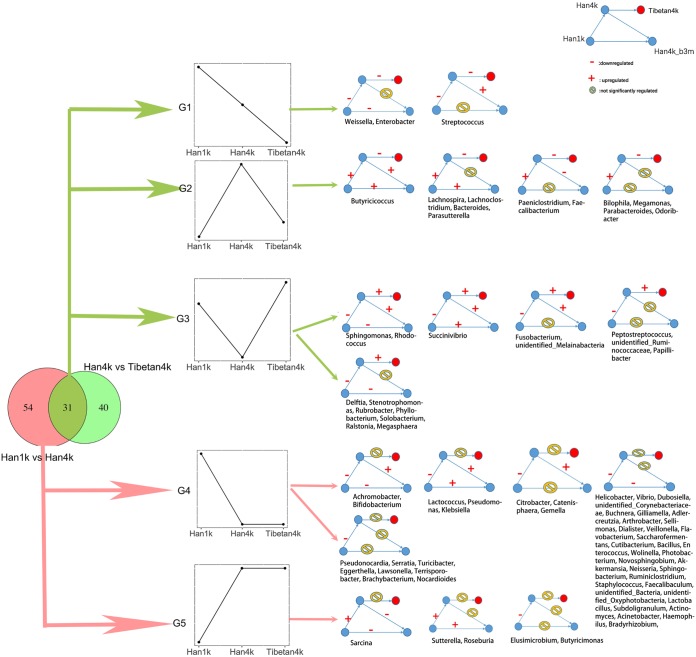
Dynamic changes in genera between different groups. The Venn diagram on the left shows the number of differentially abundant genera between Han1k and Han4k (left) and between Han4k and Tibetan4k (right) as well as the overlap between these genera. Five groups (G1 to G5), representing different change trends in the Han1k, Han4k, and Tibetan4k groups, are shown in the middle. On the right, a detailed changing trend in the Han1k, Han4k, Han4k_b3m, and Tibetan4k groups is presented with genera shown. Red dots, Tibetan4k; −, downregulated; +, upregulated; yellow circles with diagonal bars, no significant difference between two sample groups. The genera are listed in each group.

Generally, Tibetans can be considered a perfect reference population for good high-altitude adaptation. To elucidate the impact of the high-altitude environment, the dynamic changes in different genera were divided into 5 groups (G1 to G5) based on the statistically significant change trend among Han1k, Han4k, and Tibetan4k (logarithmic LDA score > 2, Wilcoxon FDR-corrected *P* value < 0.05) (Fig. 4 and genus groups sheet in Table S1). The G1 group exhibited a decreasing trend among the sequences of the plateau-living populations. The results for G4 and G5 implied that the abundance of microbes in the Han population changed significantly in response to plateau living, with a decreasing trend in the G4 group and an increasing trend in the G5 group. For the Han4k_b3m group, the level of *Butyricimonas*, a genus of a butyric acid-producing bacteria in G5, was similar to that in Han1k, suggesting that *Butyricimonas* may be a sensitive microorganism to the plateau environment. The abundance of *Roseburia* was higher in Han4k and Han4k_b3m than in Han1k, similar to the abundance in Tibetan4k. Increased abundance of *Roseburia* was found to be associated with weight loss and reduced glucose intolerance in mice (12).

### Functional alteration of the gut microbiota by the high-altitude environment.

**FIG 5:**
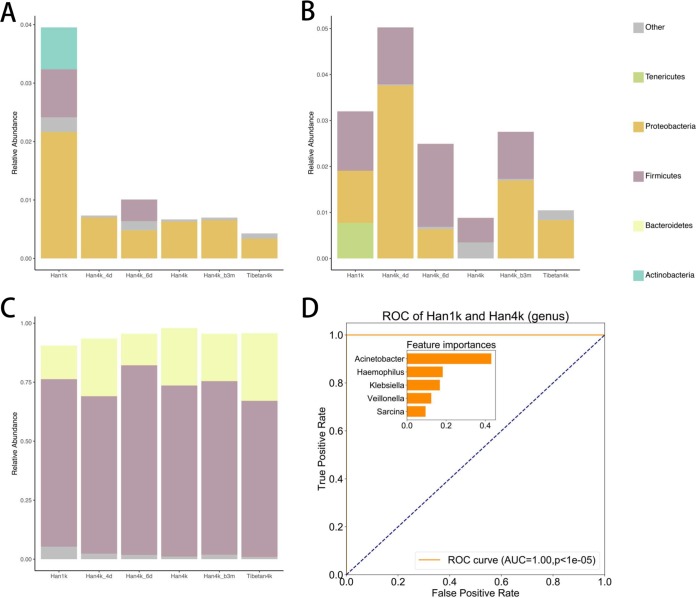
BugBase functional analysis of the gut microbiota in each group. (A) Aerobic bacterial composition in each group. (B) Facultative anaerobic bacterial composition in each group. (C) Anaerobic bacterial composition in each group. (D) Performance of the classification of Han1k and Han4k in the test data set and feature importance using an XGBoost model. AUC was used to evaluate the performance and a permutation test was used to obtain the *P* value.

The functional annotation of oxygen utilization using BugBase was compared among the groups, and a trend was observed in the proportions of aerobic, facultative anaerobic, and anaerobic bacteria with different durations of plateau living. The relative abundance distribution of the phyla in each group in the BugBase analysis is shown in Fig. 5, and the results of the Wilcoxon statistical test are shown in BugBase sheet in Table S1. Compared with that in the Han1k group, the proportion of aerobic bacteria in the Han4k group was slightly reduced (FDR-corrected *P* value = 0.08), and the Tibetan4k group maintained the lowest abundance (Fig. 5A). Most of the aerobic bacteria in the Han1k group originated from the phyla *Actinobacteria*, *Firmicutes*, and *Proteobacteria*, while the gut aerobic bacteria in the plateau-living Han groups belonged mainly to *Proteobacteria*, similarly to the Tibetan4k group (Fig. 5A).

Compared with that in Han1k, the abundance of facultative anaerobic bacteria greatly decreased in Han4k (FDR-corrected *P* value = 4.15 × 10^−6^) and was recovered in Han4k_b3m (FDR-corrected *P* value = 0.101) (Fig. 5B and BugBase sheet in Table S1). More specifically, the compositions of facultative anaerobes in Han4k and Tibetan4k were largely different. Some facultative anaerobic bacteria from *Tenericutes* appeared in only the Han1k group. The relative abundance of anaerobes, composed mainly of *Firmicutes* and *Bacteroidetes*, was higher in Tibetan4k and Han4k groups than in Han1k (FDR-corrected *P* values = 0.05 and 1.87 × 10^−7^, respectively) (Fig. 5C). Based on these results, we hypothesized that a high-altitude environment may be correlated with the reduction in the abundance of aerobic bacteria and the increase in the abundance of anaerobic bacteria.

### Group classifications based on machine learning models.

A classification model distinguishing different groups was constructed using an extreme gradient boosting (XGBoost) method (see the detailed pipeline in Fig. S4). From all genera, five genera were selected using a raw XGBoost model to build a refined XGBoost model. Among the 5 genera, Acinetobacter, *Haemophilus*, *Klebsiella*, and *Veillonella* were in G4, and *Sarcina* was in G5. The Han1k and Han4k groups were separated with an area under the ROC curve (AUC) of 1 (*P* value < 0.00001) in the test data set using the refined model (Fig. 5D). The mean AUC of 10-fold cross-validation was 0.99 with a standard deviation (SD) of 0.01, and the kappa score, a measure of model reliability, was 0.87, implying excellent performance of the model. Surprisingly, the refined model also classified Han1k and Tibetan4k groups with an AUC of 0.964 (*P* value < 0.00001) (Fig. S4). The classification performance for the Han4k and Tibetan4k groups based on the model was not as good as that for the other groups (AUC, 0.38), implying that the model was robust and that the selected genera were affected by the high-altitude environment, instead of the race differences. On the other hand, seven genera from G4 (*Odoribacter*, *Megamonas*, *Lachnospira*, unidentified *Ruminococcaceae*, *Lachnoclostridium*, *Succinivibrio*, and *Bacteroides*) distinguished Han4k and Tibetan4k with an AUC of 0.99 (*P* value < 0.00001) and a 10-fold cross-validation AUC of 0.97 with an SD of 0.1 (Fig. S4).

10.1128/mSystems.00660-19.4 FIG S4 (A) Machine learning pipeline for classifying groups using the microbiota data. (B) Performance of Han1k and Tibetan4k classification in the test dataset and feature importance in the XGBoost model pretrained using data in Han1k and Han4k. AUC was used to evaluate the performance, and a permutation test was used to obtain the *P* value. (C) Performance of Han4k and Tibetan4k classification in the test dataset and feature importance in the XGBoost model (same as for panel B). Download FIG S4, TIF file, 1 MB. Copyright © 2020 Jia et al. 2020 Jia et al. This content is distributed under the terms of the Creative Commons Attribution 4.0 International license.

### Variation in the clinical indexes indicated the influence of high altitude on Han individuals.

**FIG 6:**
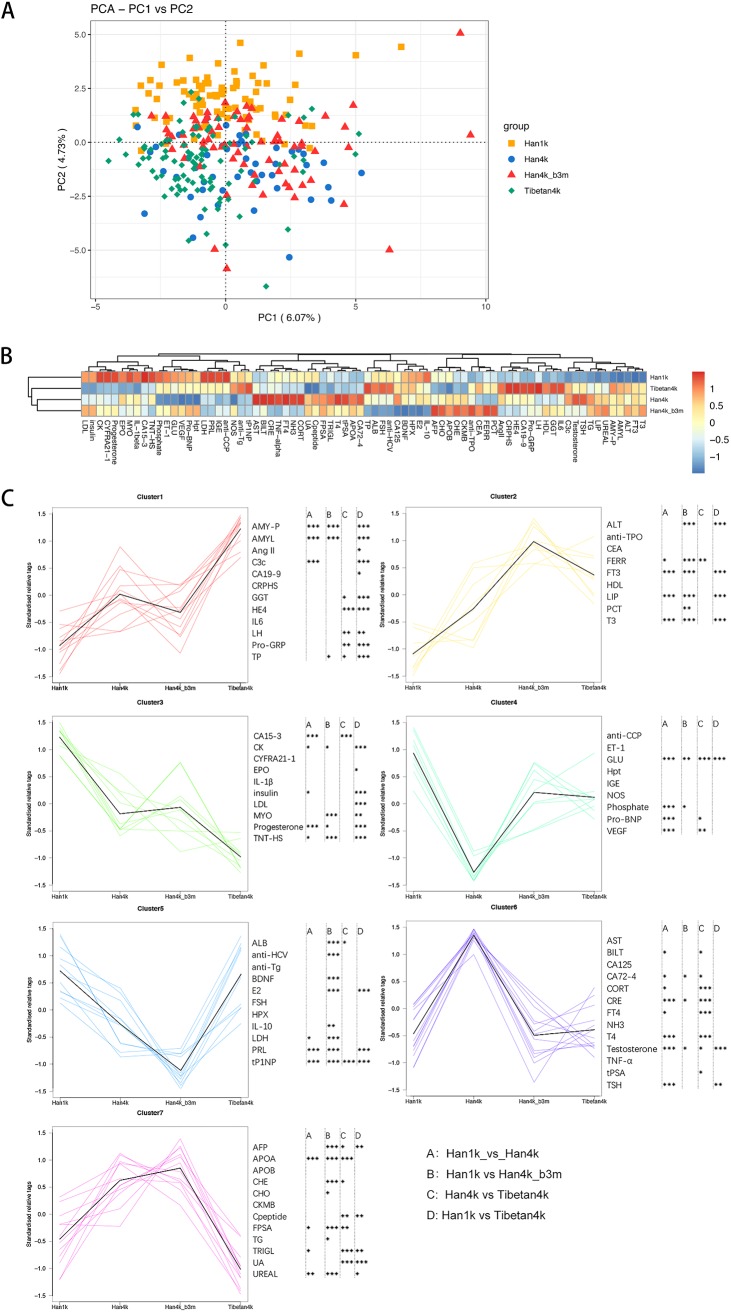
PCA and heat map of the groups based on the clinical parameters and the trend for clinical parameters among the Han1k, Han4k, Han4k_b3m, and Tibetan4k groups. (A) PCA plot of the Han1k, Han4k, Han4k_b3m, and Tibetan4k groups based on all the clinical parameter data. (B) Heat map and hierarchical clustering of the groups based on all the clinical parameter data. Groups are shown on the *y* axis, while clinical parameters are shown on the *x* axis. (C) Trends among the groups based on the clinical indexes; k-means was used to cluster the clinical parameters. Han1k, Han4k, Han4k_b3m, and Tibetan4k are shown on the *x* axis accordingly, while the scaled abundance of clinical parameters is shown on the *y* axis. The Wilcoxon statistical significance is annotated on the right: A (comparison between Han1k and Han4k), B (comparison between Han1k and Han4k_b3m), C (comparison between Han4k and Tibetan4k), and D (comparison between Han1k and Tibetan4k). *, *P* < 0.05; **, *P* < 0.01; ***, *P* < 0.001.

To test the changes in clinical indexes caused by plateau living and different living durations, 76 clinical indexes were generated from blood biochemistry measurements and enzyme-linked immunosorbent assays (ELISAs). Han1k and the remaining groups were clearly distinguished by principal-component analysis (PCA) (Fig. 6A and S5). Samples in the Han4k_b3m group were scattered among the Han4k and Tibetan4k groups in the PCA graph, suggesting that the Han4k_b3m group individuals with plateau experience maintained a signature of high altitude even after 3 months of plain living. The hierarchical clustering using all the clinical indexes showed that Han4k was close to Tibetan4k and Han4k_b3m instead of Han1k (Fig. 6B and S5).

10.1128/mSystems.00660-19.5 FIG S5 (A) PCA graph (PC1 and PC2) of the clinical indexes in all the groups. (B) PCA graph (PC2 and PC3) of the clinical indexes in all the groups. (C) Heat map and hierarchical clustering of 6 groups based on all clinical parameter data. Groups are shown on the *y* axis, while clinical parameters are shown on the *x* axis. Box graphs of each group showing the abundances of GLU (D), testosterone (E), tP1NP (F), and TRIGL (G). Download FIG S5, TIF file, 0.8 MB. Copyright © 2020 Jia et al. 2020 Jia et al. This content is distributed under the terms of the Creative Commons Attribution 4.0 International license.

The k-means clustering of the clinical indexes showed changing trends among different groups. The clinical indexes in each cluster and the statistical significance between groups are shown in Fig. 6C. Indexes in cluster 1, such as for AMYL, were related to stomach and intestine, and clusters 2 and 6 included 6 thyroid-related indexes. Several indexes in cluster 3 were related to oxygen and energy. Cluster 4 included 4 heart-related indexes: ET-1, NOS, Pro-BNP, and vascular endothelial growth factor (VEGF). Cluster 5 included 3 gonadal hormones, and cluster 7 was related to heart, kidney, and blood lipids.

The differential analysis of 76 clinical indexes among the Han1k, Han4k, and Tibetan4k groups obtained 4 parameters: GLU, testosterone, triglycerides (TRIGLs), and total procollagen type 1 amino-terminal propeptide (tP1NP) (see the clinical indexes sheet in Table S1 and Fig. S5). Overall, Han individuals on the plateau had lower GLU levels than Tibetans. All the plateau-living individuals, including Han and Tibetans individuals, had lower GLU levels than the Han1k group. This trend continued in individuals until 3 months after they returned to the plain (Han4k_b3m). It suggested that Han4k had a relatively weak TRIGL metabolism compared to that for Tibetan4k (clinical indexes sheet in Table S1). Previous studies suggested that elevated serum testosterone levels may be involved in hypoventilation and may result in CMS (13, 14).

To determine the main influence of long-term plateau life on the health status of Han individuals, XGBoost analysis was performed, and 10 clinical parameters were selected: GLU, lipase (LIP), serum testosterone levels (testosterone), ferritin (FERR), progesterone, VEGF, myoglobin (MYO), human interleukin-1β (IL-1β), bilirubin total 2,5-dichlorophenyl diazonium (DPD) (BILT), and α-amylase EPS pancreatic (AMY-P); these parameters were significantly different between Han1k and Han4k. The XGBoost-based model clearly distinguished the Han1k and Han4k groups with an AUC of 1 (*P* value = 4.4 × 10^−5^) (Fig. S6). The kappa score was 0.93, and the mean AUC of 10-fold cross-validation was also 1. Furthermore, the model also clearly distinguished the Han1k and Tibetan4k groups with an AUC of 0.90 (*P* value = 0.00005) (Fig. S6). The results suggest that these indicators may be closely related to plateau living.

10.1128/mSystems.00660-19.6 FIG S6 (A) Performance in the test data set of Han1k and Han4k classification and feature importance in the XGBoost model using clinical index data. (B) Performance in the test data set of Han1k and Tibetan4k classification and feature importance in the model trained using clinical data of Han1k and Han4k. Download FIG S6, TIF file, 0.5 MB. Copyright © 2020 Jia et al. 2020 Jia et al. This content is distributed under the terms of the Creative Commons Attribution 4.0 International license.

Similar to GLU, the level of VEGF was significantly decreased in the plateau-living Han groups and was lower than in Tibetan4k. As previously reported, VEGF stimulation was involved in restoring the oxygen supply to tissues under hypoxic conditions (15). The levels of LIP were decreased at the early stage of plateau-living (Han4k_4d or Han4k_6d), increased significantly at the steady stage (Han4k), and finally stabilized in Han4k_b3m. The level of LIP in Han4k_b3m was similar to that in Tibetan4k and higher than in Han1k.

### Correlations between clinical indexes and the gut microbiota.

**FIG 7:**
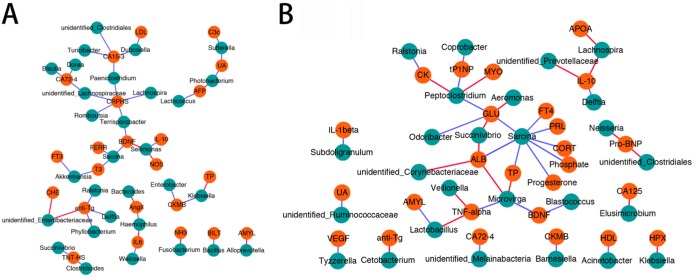
Clinical indexes and gut microbiota networks in Han1k and Tibetan4k. Only connections between clinical indexes and the gut microbiota are shown in the networks. (A) Clinical indexes and gut microbiota network in Han1k. (B) Clinical indexes and gut microbiota network in Tibetan4k. Cyan dots, bacteria; orange dots, clinical parameters; blue connecting lines, negative correlations; red connecting lines, positive correlations.

To explore the relationship between clinical indicators and the gut microbiota, we performed a Spearman univariate correlation analysis between clinical indexes and the genera (relative abundance > 0.01) in the gut with the coefficient *R* of >0.3 and *P* value of <0.05. Cytoscape was used to visualize the correlation network of Han1k and Tibetan4k (Fig. 7 and Spearman sheet in Table S1); the results of Han4k and Han4k_b3m are listed in Fig. S7. The correlation heat map between all the clinical indexes and genera is shown in Fig. S8. Unlike the Han1k network (Fig. 7A), *Sarcina* and GLU were the hub nodes in the Tibetan4k network (Fig. 7B). Additionally, *Succinivibrio* and *Aeromonas* were positively correlated with GLU (*r* = 0.44 and 0.31, respectively), while *Sarcina*, *Odoribacter*, and *Peptoclostridium* were negatively correlated with GLU (*r* = −0.32, −0.32, and −0.36, respectively). *Coprobacter* and *Peptoclostridium* were negatively correlated with tP1NP (*r* = −0.30 and −0.36, respectively). For the Han4k group, the clinical parameters and gut microbiota were closely associated, unlike for the other groups (Fig. S7).

10.1128/mSystems.00660-19.7 FIG S7 (A and B) Clinical indexes and gut microbiota networks in Han4k and Han4k_b3m. Cyan dots, bacteria; orange dots, clinical parameters; blue connecting lines, negative correlations; red connecting lines, positive correlations. Download FIG S7, TIF file, 1.6 MB. Copyright © 2020 Jia et al. 2020 Jia et al. This content is distributed under the terms of the Creative Commons Attribution 4.0 International license.

10.1128/mSystems.00660-19.8 FIG S8 (A to D) Heat maps of the clinical indexes and bacteria in the Han1k, Han4k, Han4k_b3m, and Tibetan4k groups. The sizes of the circles represent the extent of correlation; red, negative correlation; blue, positive correlation. Download FIG S8, TIF file, 1.8 MB. Copyright © 2020 Jia et al. 2020 Jia et al. This content is distributed under the terms of the Creative Commons Attribution 4.0 International license.

**FIG 8:**
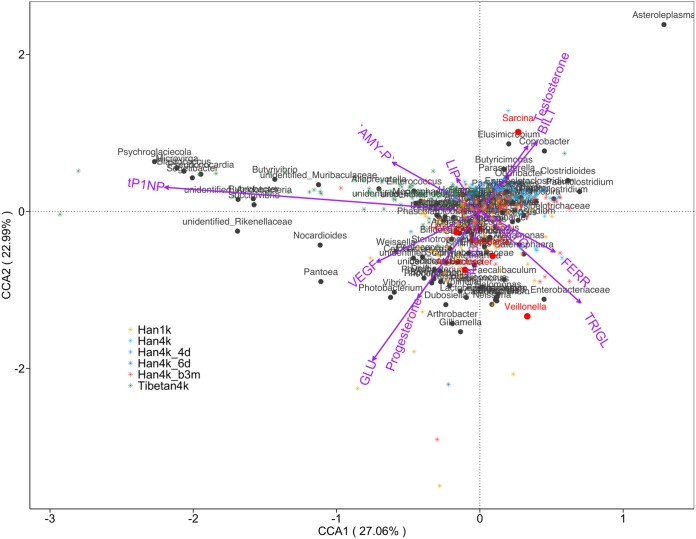
Canonical correspondence analysis of the clinical indexes and bacterial composition. The graph shows the relationships among samples (stars), clinical indexes (vectors), and bacteria (dots). The colors of the stars represent sample groups. The segment length of each blood clinical index indicates the strength of the association between the variable and the microbial community. Bacteria indicated by red dots were used in the XGBoost-based classification of Han1k and Han4k.

The relationship between the clinical indexes and microbial community was discerned by canonical correspondence analysis (CCA) (Fig. 8). The Han1k, Han4k, and Tibetan4k groups were clearly distinguished, consistent with the PCoA. The clinical parameters GLU, VEGF, and progesterone were correlated with and linked to the Han1k group, tP1NP and AMY-P were correlated with and linked to the Tibetan4k group, and BILT and testosterone were correlated with and linked to the Han4k and Han4k_b3m groups, respectively. Notably, TRIGL and FERR were negatively correlated with the Tibetan4k group. At the genus level, *Succinivibrio* and *Butyrivibrio* were related to the Tibetan4k groups, and Acinetobacter and *Veillonella* were associated with the Han1k group, while *Sarcina* was related to the Han4k groups and was negatively correlated with GLU.

## DISCUSSION

This study systematically evaluated the changes in the intestinal microbiota and the clinical indexes of the Han and Tibetan populations with different durations of living on the Qinghai-Tibet Plateau. As oxygen is essential to human beings, hypobaric hypoxia, a major characteristic of the plateau, could induce many changes in individuals living on the plateau. We showed that the high-altitude environment quickly and strongly impacts the intestinal microbiota in a directional manner and showed the clinical indexes and microbiota landscapes in the studied cohorts.

The microbiota analysis suggests that the high-altitude environment rapidly and continually influenced the gut microbiota in the Han participants. Our study shows that the intestinal microbiota of the Han population living on the plain is noticeably different from that of the Tibetan population. Compared with that in the non-plateau-living Han individuals, the intestinal microbiota of the plateau-living Han population changes to become increasingly similar to the microbiota of the Tibetan population. This finding indicates that the plateau has a significant impact on the gut microbiota and that the change in the microbiota may be related to the plateau adaptation process of the Han population. In 4 to 6 days, the gut microbiota in the Han individuals had changed, and these changes were maintained for at least 3 months in the plateau-living Han individuals and even in the Han individuals on the plain who had returned from the plateau 3 months prior. This finding suggests that the influence of plateau life on the microbiota is rapid, consistent, directional, and persistent. These findings were confirmed by analysis of different characteristics of the microbiota, including the α-diversity, β-diversity, bacterial composition at different taxonomic levels, and function.

Apparently, hypoxia had a considerable impact on the microbiota of plateau-living Han individuals, and these signatures did not recover within 3 months after leaving the plateau. Liu et al. recently reported that a Chinese volunteer team showed dynamic patterns of microbial communities across longer time scales with multiple dietary shifts and found that these participants rapidly recovered their gut microbiota after returning from Trinidad and Tobago to Beijing with dietary changes (16). The NASA twins study showed that the gut microbiota was recovered in individuals several weeks after space flight, compared with that observed preflight (17). However, this phenomenon did not occur in the Han4k_b3m cohort in our study. This finding indicates that the gut microbiota probably requires a long recovery period after experiencing a severe and lasting hypoxic environment, as oxygen is essential to humans. Overall, our study showed that the high-altitude environment played an important role in shaping the gut microbiota in Han individuals who were on the plateau or had been to the plateau.

From the microbiota analyses, including the β-diversity analysis, the UPGMA analysis based on the bacterial composition, the bacterial function analysis based on the BugBase annotations, and the clinical indexes we found that Han4k_4/6d was closer to Han4k, Han4k was closer to Tibetan4k, and Han4k_b3m was closer to Han4k than Han1k. It was reported that a switch from aerobic to anaerobic metabolism and increase in the basal metabolic rate occurred after HIF-1 activation on the plateau (15). These results indicate that the metabolism in the gut microbiota of Han individuals on the plateau was perturbed due to the high-altitude environment.

We found that the mechanisms of adaptation to the plateau environment in the Han and Tibetan individuals were different. There remained differences in the microbiota in plateau-living Han individuals and native Tibetans. More importantly, individuals at high altitude, including those in the Han4k_4d, Han4k_6d, Han4k, and Tibetan4k groups, had lower GLU levels than those in Han1k. Notably, the low GLU levels in individuals living at high altitudes was previously discovered in plasma samples of Andean populations (11). However, there are no previous reports regarding Tibetans in Tibet, China. Because blood glucose levels are regulated by insulin, we also investigated the levels of insulin in different groups and found that the insulin level is associated with the decreased GLU levels, as the insulin level was relatively low in participants on the plateau. Studies have also demonstrated that hyperinsulinemia is not a cause of decreased glycemia (11). We argue that there should be other factors responsible for the decreased GLU levels in participants on the plateau. Acclimatization to high altitude appears to involve the consumption of large amounts of blood GLU in human skeletal muscles and heart muscles (11). Decreased fasting glycemia can be acquired by the Han population on the plateau, probably as an adaptation to the high-altitude environment, as the Tibetan population also had a lower GLU level than the Han1k group. Both Tibetan4k and Han4k had low GLU levels, while Tibetan4k had a lower TRIGL level than Han1k, and Han4k had a higher TRIGL level than Han1k. Therefore, we argue that while high energy consumption occurred on the plateau, the mechanisms differed between the Han and Tibetan individuals.

A Tibetan4k-specific connection between GLU and bacteria was identified. It was reported that the human gut microbiome impacts the serum metabolome (18). *Succinivibrio*, which is enriched in rumen microbial ecosystems, can efficiently ferment glucose, producing acetic and succinic acids, and metabolize various fatty acids, which may increase energy utilization efficiency (19, 20). Notably, *Sarcina* can produce cellulose. One species in the *Odoribacter* genus can produce butyrate and modulate the host’s blood glucose concentration (21–23). *Peptoclostridium* can produce beta-phosphoglucomutase, participating in starch and sucrose metabolism. These bacteria are closely associated with the regulation of GLU levels in Tibetans, which probably play an important role in adaptation to high altitudes.

In summary, the high-altitude environment has a profound impact on the gut microbiota and blood clinical indexes of Han and Tibetan individuals, reflecting the different adaptations to high-altitude among individuals. We plan to perform a follow-up cohort study on the same participants using a multiomics approach to obtain a deeper understanding and further expand our conclusion. We will evaluate the high-altitude adaptivity of the Han population on the plateau to illustrate their different mechanism of adaptation to the high-altitude environment and identify a drug target for mountain sickness.

### Conclusions.

Our results provide further evidence that the high-altitude environment has a considerable influence on the gut microbiota and the blood clinical indexes of Han individuals, who usually do not live on the plateau. Importantly, for the first time, we found that the individuals who lived on the plain for 3 months after returning from the plateau did not recover to their nonplateau status in terms of the ɑ-diversity, β-diversity, composition and functional capabilities of the gut bacteria, blood clinical indexes, the subspace projected by the bacteria, and the clinical indexes determined via CCA. Compared with the Han population on the plain, the Han population on the plateau were closer to the Tibetans in terms of microbiota signature, clinical indexes, and projected subspace. However, the mechanisms of adaptation to the plateau environment in the Han and Tibetan populations were different. There were Tibetan-specific correlations between blood GLU levels and *Succinivibrio* (positive) and *Sarcina* (negative) abundance in the intestine. This study will improve our understanding of the impact of the hypoxic environment on the gut microbiota and blood clinical indexes as well as the adaptation mechanism and intervention targets for plateau adaptation.

## MATERIALS AND METHODS

### Study cohort.

All study protocols were approved by the ethics committee of the Chinese PLA General Hospital with the approval identifier (ID) S2019-035-01 and were in accordance with established national and institutional ethical guidelines. The study was clearly described to all participants, who signed informed consent forms before the collection of blood, fecal samples, and personal information. We carefully designed the experiment and the enrollment criteria to largely eliminate differences due to important factors, such as diet and lifestyle, in the microbiome study. A total of 393 healthy Han and Tibetan Chinese residents who lived on the plain or on the plateau were recruited for this study and divided into six groups according to differences in plateau living duration (Han1k, Han4k_4d, Han4k_6d, Han4k, Han4k_b3m, and Tibetan4k). Participants in the Han1k group worked in Xinjiang, China, at an altitude of 1,200 m. They had never been to the plateau previously. Han4k_4d and Han4k_6d individuals stayed in Tibet, China, at an altitude of 4,300 m, for 4 or 6 days during sampling (the first plateau experience). They had never been to a plateau previously. Han4k individuals worked in Tibet, at an altitude of 4,300 m, for more than 3 months and had never been on the plateau previously. Han4k_b3m individuals went to the plateau for more than 3 months and then stayed on the plain for 3 months for skill training after the unique plateau experience. All the samples from plateau-living participants were collected on the plateau at the time point indicated, while samples from Han1k and Han4k_b3m were obtained on the plain.

The diets and lifestyles of the participants on the plateau, including Tibetans, were largely similar, as they had traditional Chinese food (rice, wheat, vegetable oil, meat, and fish) in the employer’s canteens and lived in the employer’s dormitories. Nonplateau participants had similar diets and lifestyles. Accordingly, the design largely eliminated several confounding factors (diet and lifestyles), and the main factor was the high-altitude hypoxic environment. The participants had no known clinical diseases and did not take medication or receive any intervention before the ascent to high altitude and on the plateau. Metadata statistics are listed in Table S1 in the supplemental material.

### Collection of blood and fecal samples, biochemistry tests, and ELISAs.

Morning fasting venous blood (8 ml) was collected with EDTA-K2. Blood samples were centrifuged at 4,000 rpm for 10 min to separate plasma and stored at −80°C until used in assays in our hospital (Beijing, an altitude of approximately 40 m). Two milliliters of plasma was used to assay 76 clinical test items using a hematology analyzer (cobas 6000; Roche, USA). In addition, 1.5 ml of plasma was used in 11 ELISAs (Expand Bio, China). All blood test items and basic statistical descriptions are listed in the clinical indexes sheet in Table S1.

Fresh stool samples were collected with a sterile manure collector. The middle part was obtained with a sterile fecal sampler. Approximately 0.2 g of each stool sample was weighed in a sterile centrifuge tube and aliquoted to a new 1.5-ml Eppendorf tube. Each sample was transferred for storage at −80°C within 20 min.

### 16S rRNA gene sequencing.

DNA extraction from the stool samples and PCR amplification of the V3 to V4 region of the 16S rRNA genes were performed with a standard protocol described by Novogene (Beijing). Equal volumes of 1× loading buffer (containing SYBR green) and amplified PCR products were mixed and used to generate sequencing libraries using the Ion Plus Fragment Library kit 48 rxns (Thermo Scientific, USA) according to the manufacturer’s protocol. High-throughput sequencing of the library was performed on an Ion S5 XL platform, and approximately 400-bp single-end raw reads were produced per sample.

### Data analysis and quality control.

Quality filtering of the raw reads was performed, and high-quality clean reads were obtained according to the quality control process of cutadapt (v1.9.1) software (24). The reads were compared with the SILVA reference database (release132) (25) by USEARCH software (26) to detect and remove chimera sequences.

Sequences with ≥97% similarity, as determined by USEARCH software, were assigned to the same OTUs. A representative sequence for each OTU was screened for further annotation. Each representative sequence was annotated with the SILVA database based on the Mothur algorithm to obtain the taxonomic information for each OTU. To study the phylogenetic relationships of different OTUs and the differences in the dominant genera in different groups, multiple-sequence alignment was conducted using USEARCH software.

Rarefaction curve analysis was used to judge whether the sequencing data were adequate to characterize the bacterial richness of all the samples. OTU abundance was normalized by rarefying each sample such that all the samples had the same number of total counts (60,887 reads). Subsequent analyses of α-diversity and β-diversity were performed based on this normalized data output. The α-diversity was applied to analyze the complexity of bacterial diversity for a sample through 4 indexes, including Chao1, Shannon, Simpson, and ACE. All these indexes were calculated with QIIME (v1.7.0) (27) and visualized with R software (version 2.15.3). Two indexes were selected to identify community richness, namely, the Chao1 and Shannon indexes. The β-diversity analysis was used to evaluate differences in bacterial complexity of the samples. β-Diversity on both weighted and unweighted UniFrac distances was calculated via QIIME software. PCoA was performed to obtain principal coordinates from complex multidimensional data and to visualize them. Adonis analysis was performed among all the groups via the R package vegan. A UPGMA hierarchical clustering tree, constructed by QIIME, was examined to interpret the distance matrix using average linkage.

BugBase was used for functional annotation of the microbiota. The OTU table, annotated with the Greengenes 97% OTU reference data set, was standardized according to the predicted 16S copy number; then, the microbiological phenotype was pretested on the basis of the prepossessed database and the threshold of the BugBase tool to automatically select the threshold of the microorganism. The threshold of the *P* value (no FDR correction) was 0.05, and the logarithm of LDA score was set to greater than 2 in the LEfSe analysis (28). Meanwhile, the Wilcoxon statistical test with Benjamini-Hochberg correction was used to obtain the differentially abundant bacteria with an FDR-corrected *P* value of less than 0.05. The overlapped genera between the results of LEfSe and Wilcoxon were considered the final differentially abundant bacteria between groups. The Spearman correlation (the Hmisc R package) and CCA, implemented via the R package vegan, were used to calculate the association of the clinical parameters and gut bacterial composition. For the Spearman correlation analysis, the threshold of the *P* value was 0.05, and *r* was 0.3. Analysis of the network of clinical parameters and gut bacterial composition was conducted by Cytoscape.

The python package of XGBoost was used to select features and construct models (Fig. S4). The samples were split randomly using the sklearn package. Overall, 75% of the samples in the Han4k group and 50% of samples in the Han1k group were used to train the models, and 25% of the samples in the Han4k and Han1k groups were used to test the model. To validate the model in the Han1k and Tibetan4k groups, the remaining samples of the Han1k group (25%) and all samples of the Tibetan4k group were used. AUC was used to evaluate the performance of the model in the test and validate the data. For all the models constructed, 10-fold cross-validation was also used to validate the accuracy and stability of the models. A permutation test was used to identify the statistical significance of the AUC.

### Statistical analysis.

The shapiro.test function in the R statistics package was used to perform the Shapiro-Wilk test of normality with a *P* value cutoff of 0.05 for the clinical indexes data. Independent *t* tests were used to compare the differences of normally distributed variables between two groups. The Wilcoxon test was applied to evaluate differences between nonnormally distributed data. The Benjamini-Hochberg method was used to control the FDR.

### Data availability.

The data set supporting the conclusions of this article are available in the Genome Sequence Archive repository under accession PRJCA001483.
