# Supplementary figures and images for: Impacts of the Plateau Environment on the Gut Microbiota and Blood Clinical Indexes in Han and Tibetan Individuals
Source: mSystems. 2020 Jan 21;5(1):e00660-19. doi: 10.1128/mSystems.00660-19 (PMC6977073; doi:10.1128/mSystems.00660-19)

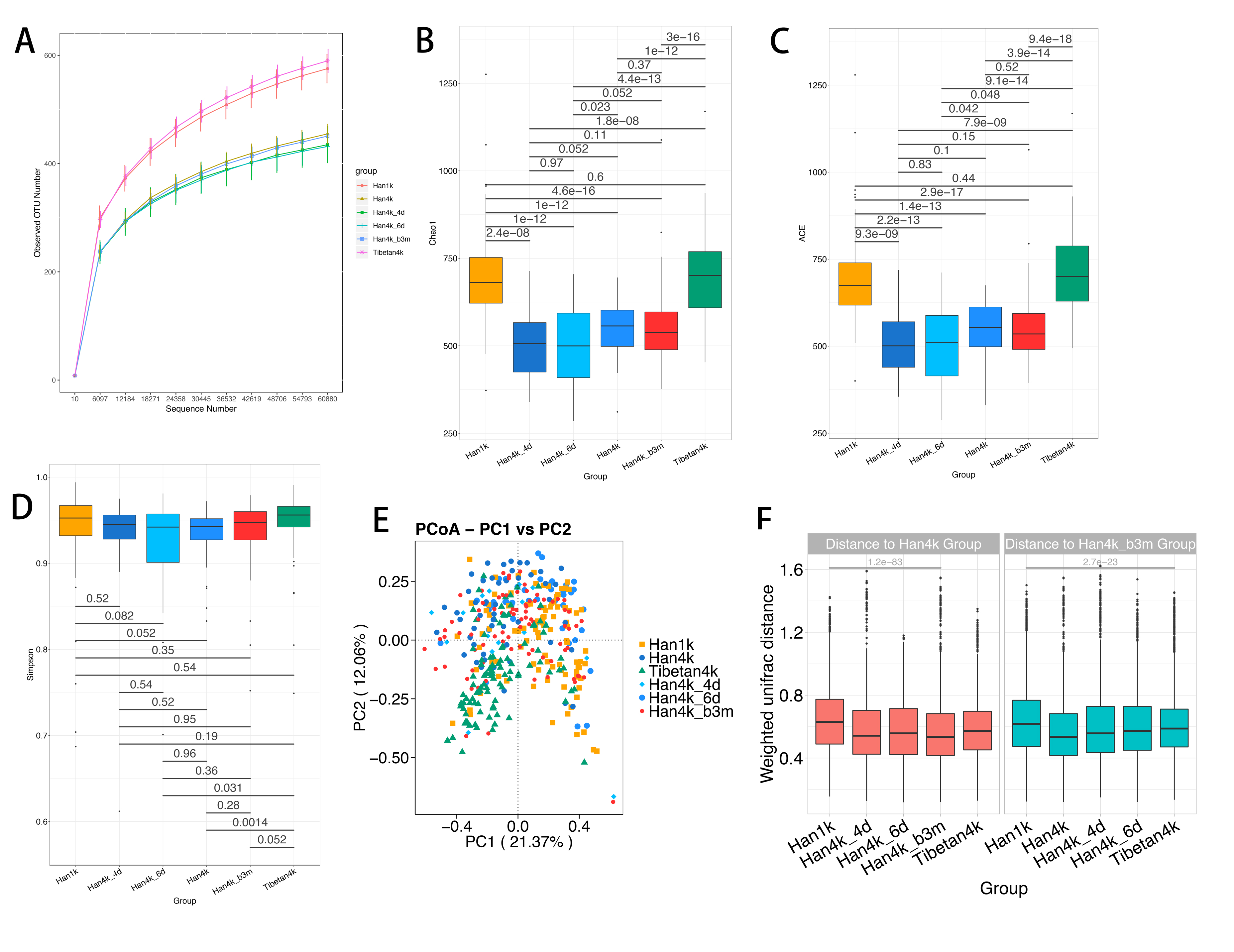

Supplement: FIG S1 [file mSystems.00660-19-sf001.tif]

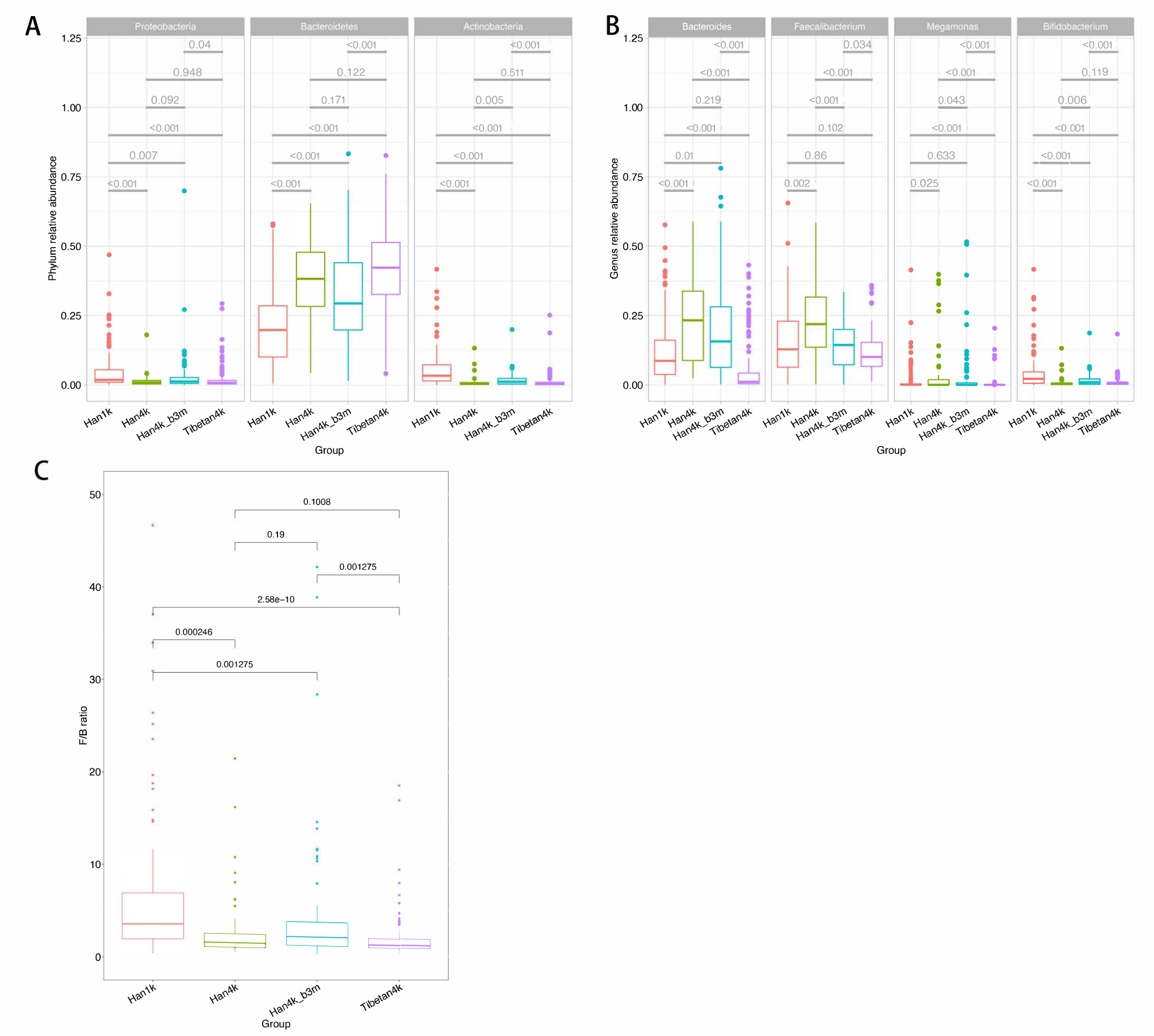

Supplement: FIG S2 [file mSystems.00660-19-sf002.tif]

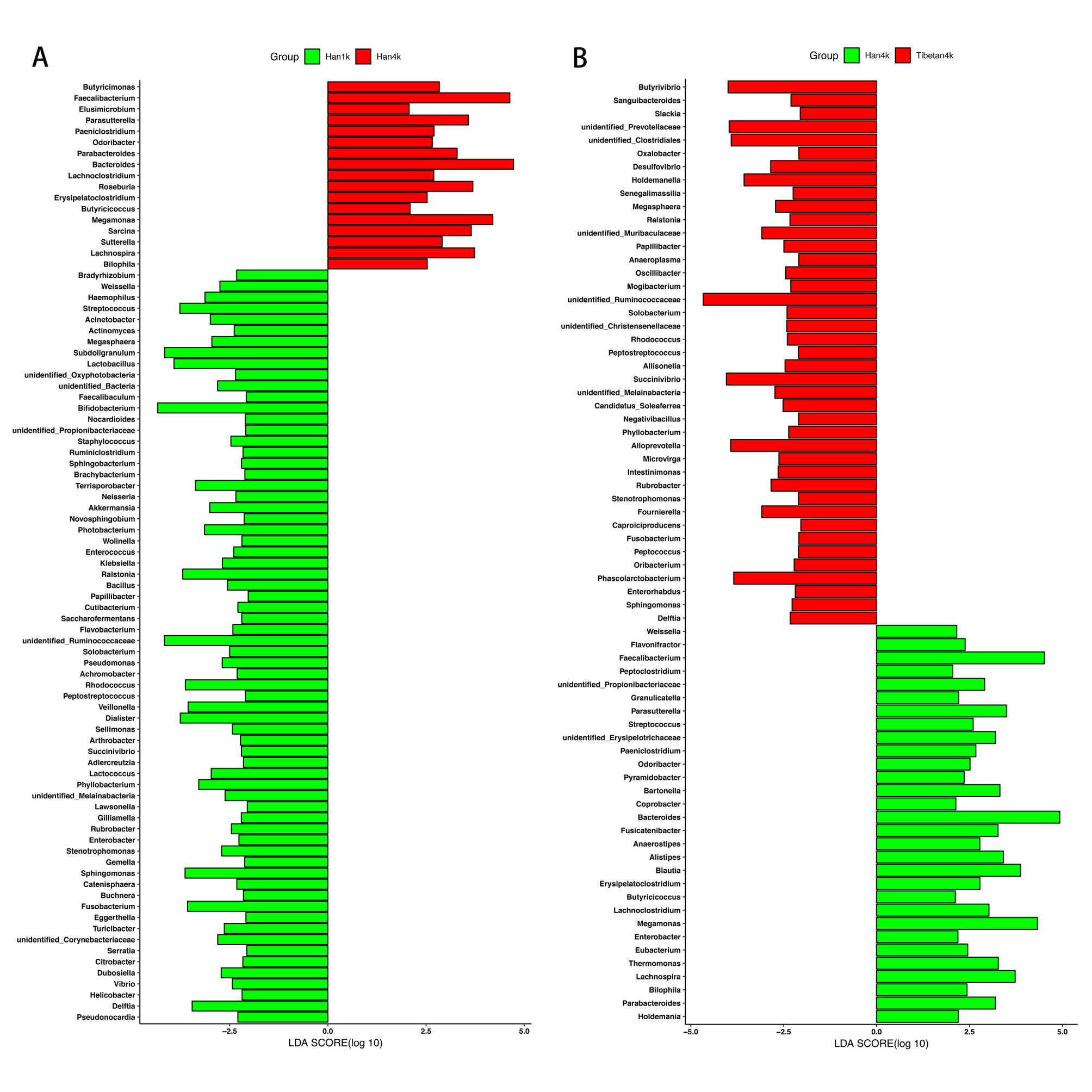

Supplement: FIG S3 [file mSystems.00660-19-sf003.tif]

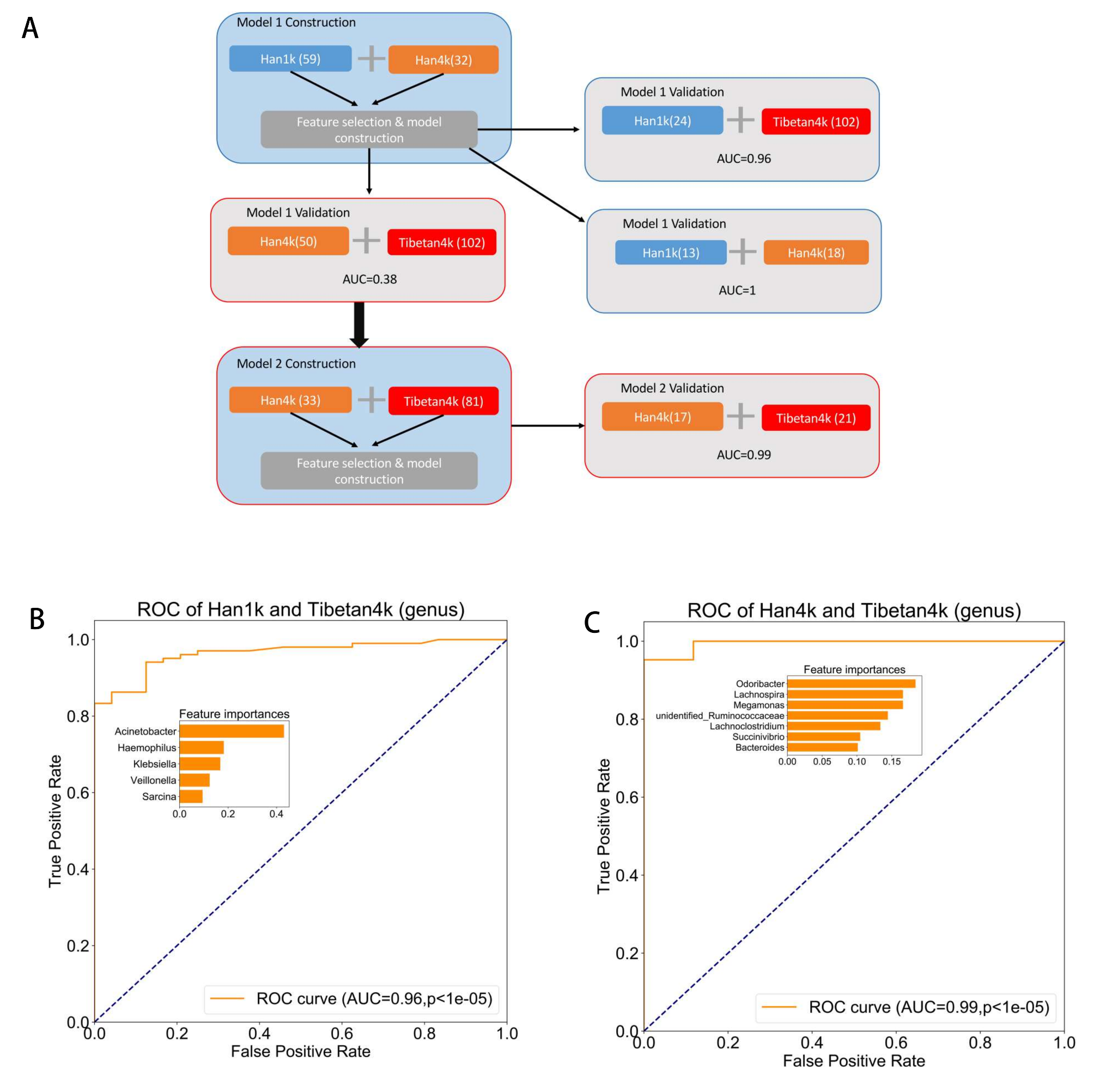

Supplement: FIG S4 [file mSystems.00660-19-sf004.tif]

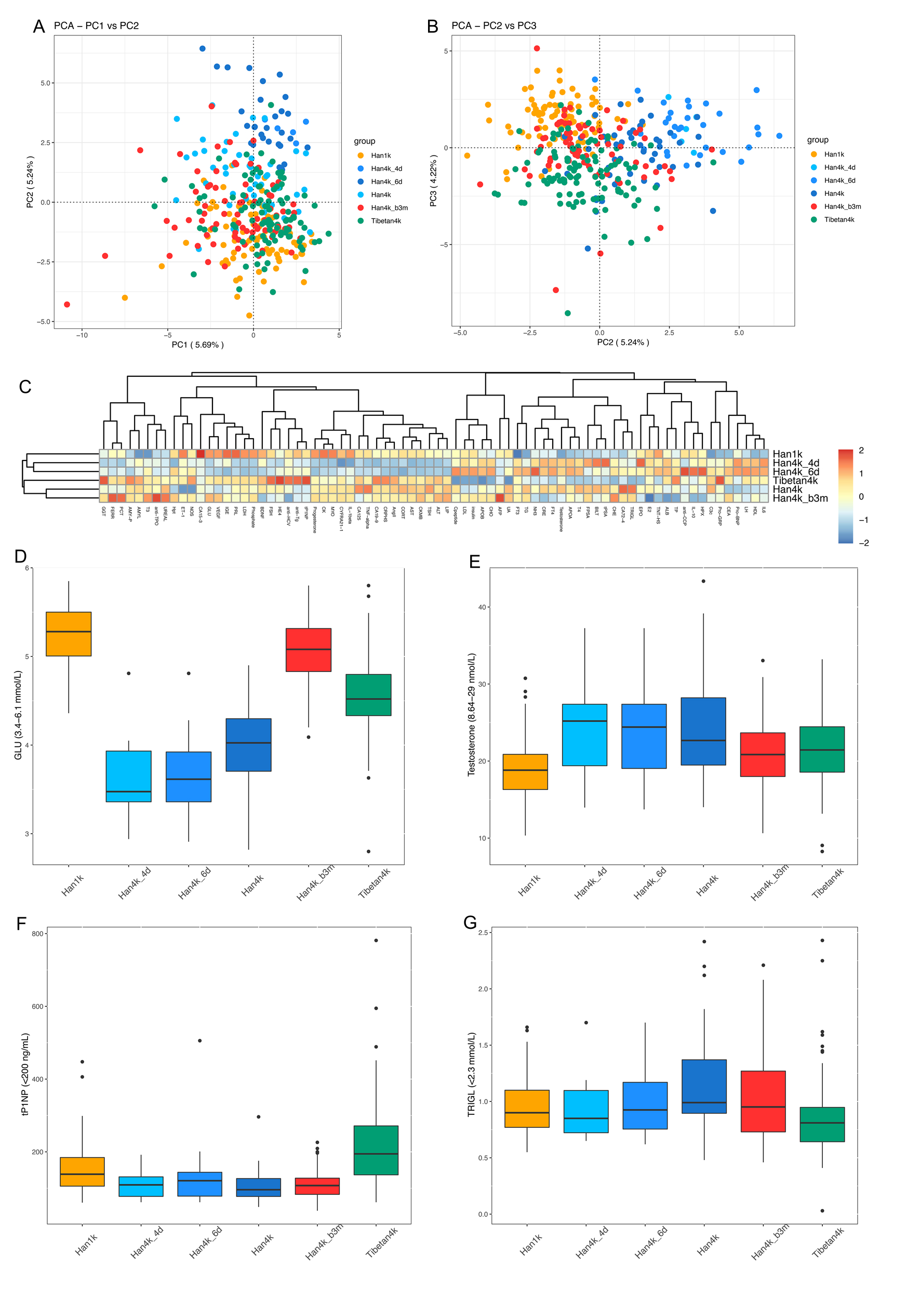

Supplement: FIG S5 [file mSystems.00660-19-sf005.tif]

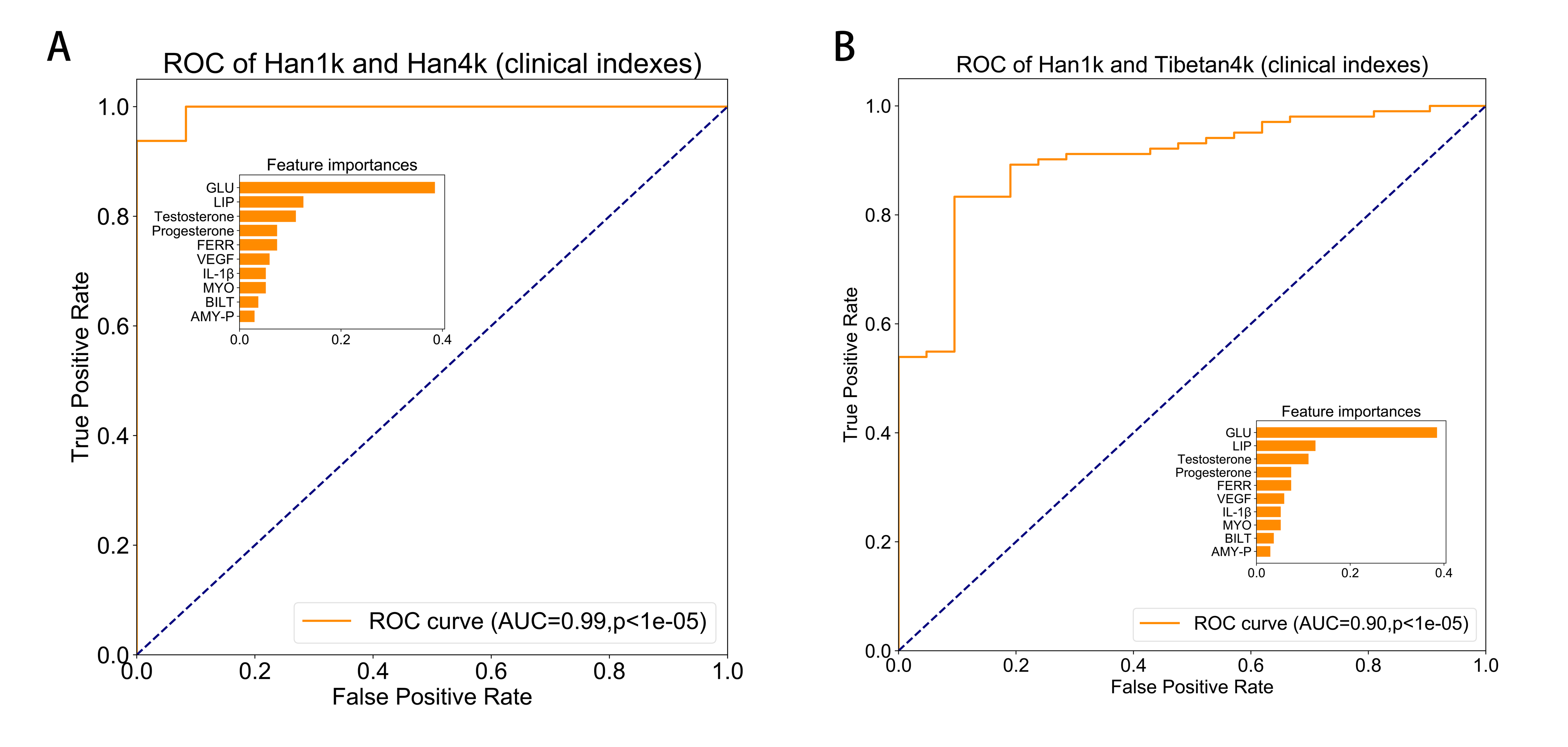

Supplement: FIG S6 [file mSystems.00660-19-sf006.tif]

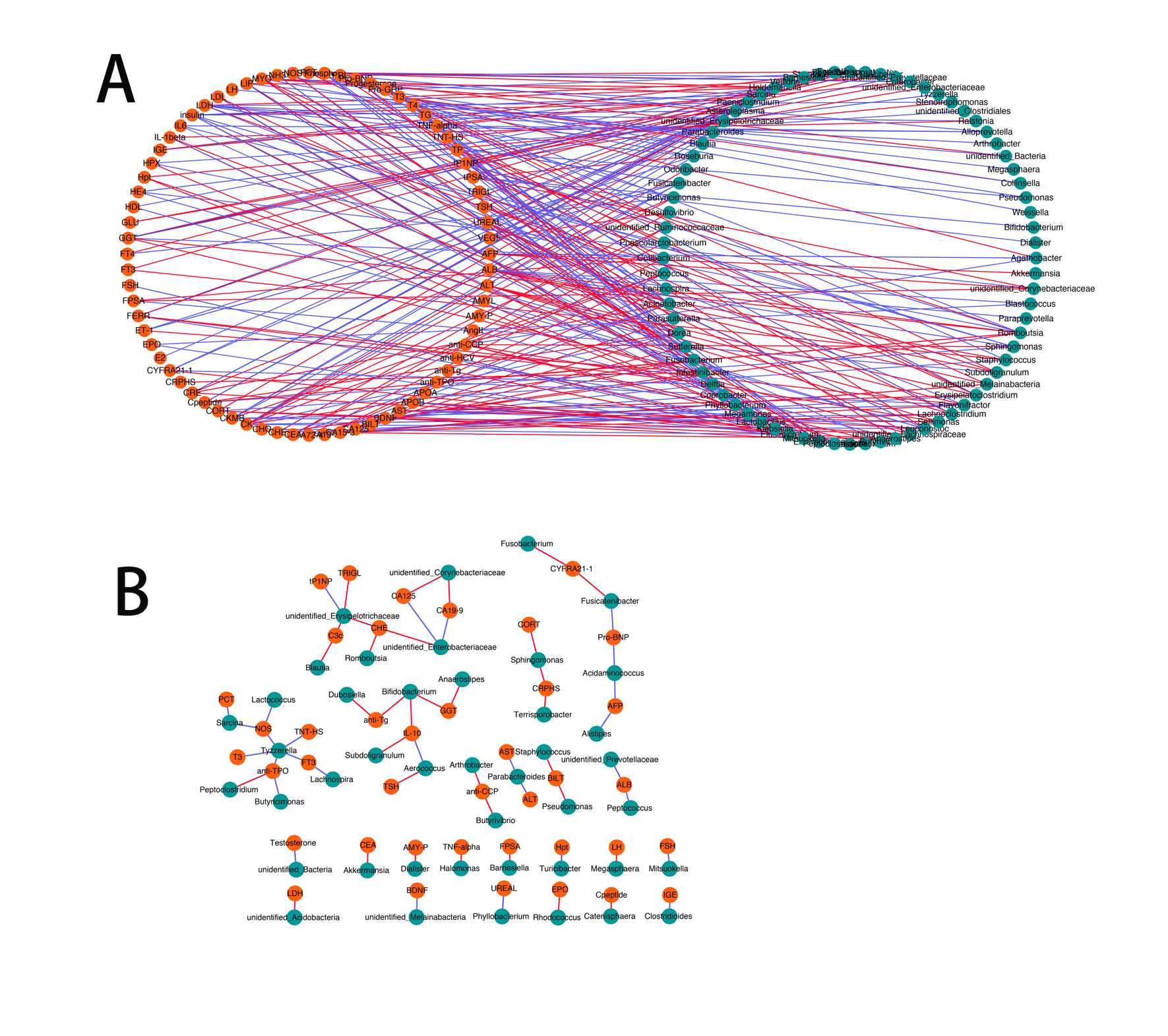

Supplement: FIG S7 [file mSystems.00660-19-sf007.tif]

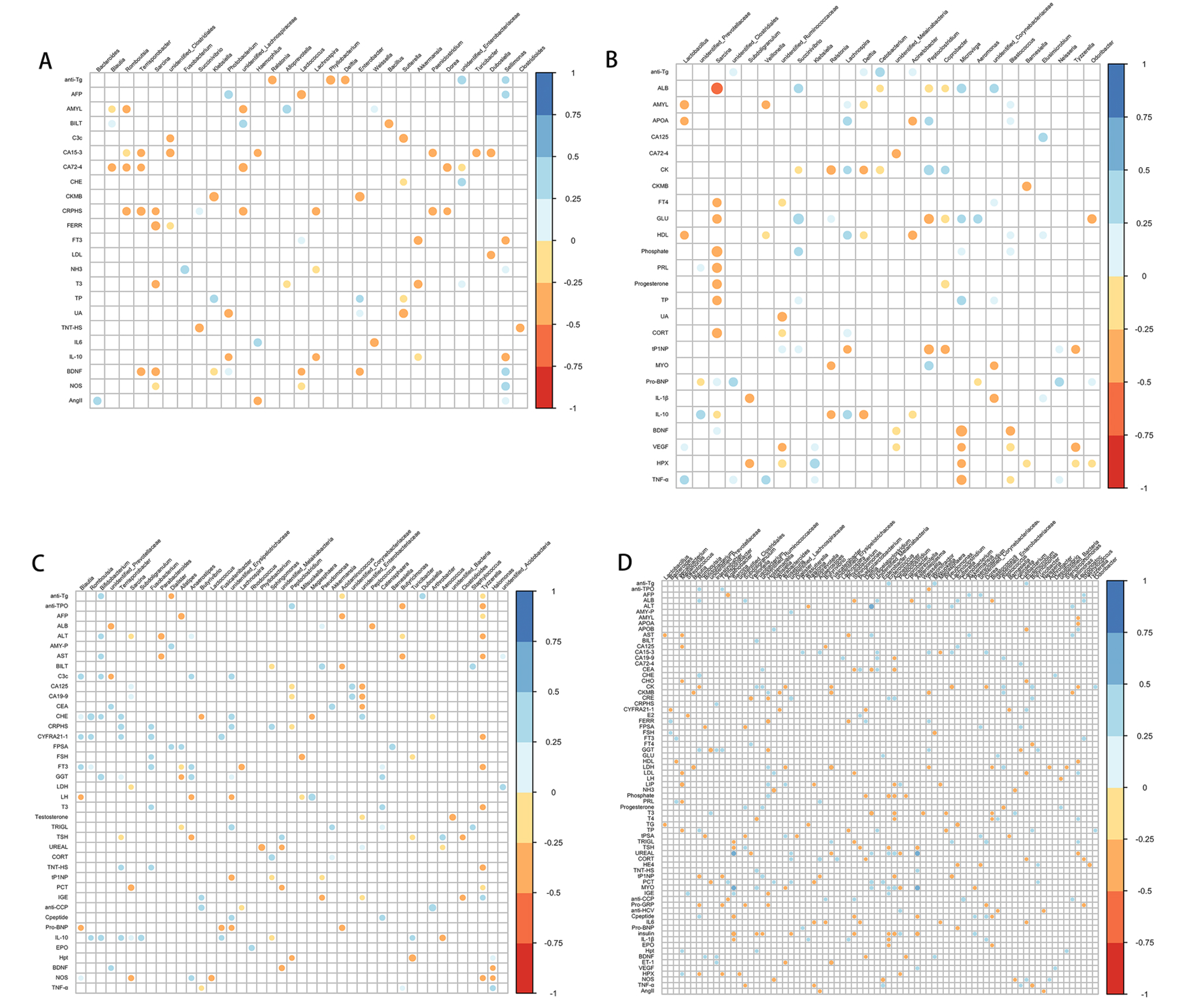

Supplement: FIG S8 [file mSystems.00660-19-sf008.tif]
